# Induction of multi-antigen multi-stage immune responses against *Plasmodium falciparum *in rhesus monkeys, in the absence of antigen interference, with heterologous DNA prime/poxvirus boost immunization

**DOI:** 10.1186/1475-2875-6-135

**Published:** 2007-10-09

**Authors:** George Jiang, Yupin Charoenvit, Alberto Moreno, Maria F Baraceros, Glenna Banania, Nancy Richie, Steve Abot, Harini Ganeshan, Victoria Fallarme, Noelle B Patterson, Andrew Geall, Walter R Weiss, Elizabeth Strobert, Ivette Caro-Aquilar, David E Lanar, Allan Saul, Laura B Martin, Kalpana Gowda, Craig R Morrissette, David C Kaslow, Daniel J Carucci, Mary R Galinski, Denise L Doolan

**Affiliations:** 1 Malaria Program, Naval Medical Research Center, Silver Spring, MD 20910-7500, USA; 2 Henry M. Jackson Foundation, Rockville, MD 20852, USA; 3 Emory Vaccine Center, Yerkes National Primate Research Center, Emory University, Atlanta, GA 30329, USA; 4 Division of Infectious Diseases, Department of Medicine, Emory University School of Medicine, Emory University, Atlanta, GA 30329, USA; 5 Vical, Inc., San Diego, CA 92121, USA; 6 Walter Reed Army Institute of Research, Silver Spring, MD 20910-7500, USA; 7 Malaria Vaccine Development Branch, National Institute of Allergies and Infectious Diseases, National Institutes of Health, Rockville, MD 20852, USA; 8 Department of Molecular Microbiology and Immunology, School of Hygiene and Public Health, Johns Hopkins University, Baltimore, MD 21205-2179, USA; 9 The Queensland Institute of Medical Research, The Bancroft Centre, 300 Herston Road, PO Royal Brisbane Hospital, Brisbane QLD 4029 Australia

## Abstract

The present study has evaluated the immunogenicity of single or multiple *Plasmodium falciparum (Pf) *antigens administered in a DNA prime/poxvirus boost regimen with or without the poloxamer CRL1005 in rhesus monkeys. Animals were primed with *Pf*CSP plasmid DNA or a mixture of *Pf*CSP, *Pf*SSP2/TRAP, *Pf*LSA1, *Pf*AMA1 and *Pf*MSP1-42 (CSLAM) DNA vaccines in PBS or formulated with CRL1005, and subsequently boosted with ALVAC-*Pf*7, a canarypox virus expressing the CSLAM antigens. Cell-mediated immune responses were evaluated by IFN-γ ELIspot and intracellular cytokine staining, using recombinant proteins and overlapping synthetic peptides. Antigen-specific and parasite-specific antibody responses were evaluated by ELISA and IFAT, respectively. Immune responses to all components of the multi-antigen mixture were demonstrated following immunization with either DNA/PBS or DNA/CRL1005, and no antigen interference was observed in animals receiving CSLAM as compared to *Pf*CSP alone. These data support the down-selection of the CSLAM antigen combination. CRL1005 formulation had no apparent effect on vaccine-induced T cell or antibody responses, either before or after viral boost. In high responder monkeys, CD4+IL-2+ responses were more predominant than CD8+ T cell responses. Furthermore, CD8+ IFN-γ responses were detected only in the presence of detectable CD4+ T cell responses. Overall, this study demonstrates the potential for multivalent *Pf *vaccines based on rational antigen selection and combination, and suggests that further formulation development to increase the immunogenicity of DNA encoded antigens is warranted.

## Background

Despite intense research efforts, malaria remains a significant public health problem [1] and is associated with significant constraints on economic progress and productivity [2] in the developing world. Especially with the spread of drug-resistant *Plasmodium *parasites and insecticide-resistant *Anopheles *vectors, development of an effective malaria vaccine is considered a public health priority [3]. Two human models demonstrate the feasibility of developing a malaria vaccine. Immunization with radiation-attenuated *Plasmodium spp*. parasites has been shown to confer sterile protection against sporozoite challenge in humans [4,5] as well as rodent [6] and non-human primate [7] models, and natural long-term exposure to the parasite is associated with an age-related decrease in the incidence, prevalence, and density of infection [8]. The critical effector mechanism in the radiation-attenuated sporozoite model is thought to be CD8+ T-cell responses directed against parasite antigens expressed in the liver stage [9-11]. In the naturally acquired immunity model, antibodies directed against blood-stage parasite antigens are thought to be responsible for protective immunity [12-14].

Based on these two models, a multi-stage multi-immune response vaccine against malaria comprising antigens expressed in the liver stage and targeted by T-cell responses, as well as antigens expressed in the blood-stage and targeted by antibody responses, is being developed [15]. The hypothesis is that by reducing the numbers of parasites emerging from the liver (T-cell immune responses directed against those antigens expressed by irradiated sporozoites in hepatocytes) and priming the immune system to erythrocytic stage antigens that will be boosted by infection from natural exposure (antibody responses directed against parasite proteins expressed on the surface of merozoites or infected erythrocytes or in apical organelles), one will reduce the severity and mortality due to *Plasmodium falciparum *malaria. This "combined stage" approach is designed to prevent infection by killing the majority of developing parasites in the liver, and also to prevent severe disease and death should break-through blood stage infections occur. This vaccine development strategy originally called for constructing vaccines consisting of plasmid cocktails of increasing valency, beginning with five pre-erythrocytic stage antigens, and then adding ten or more erythrocytic stage antigens [15]. However, a clinical trial of five pre-erythrocytic stage vaccines (*Pf*CSP, *Pf*SSP2/TRAP, *Pf*LSA1, *Pf*LSA3, *Pf*Exp1) indicated reduced immunogenicity to components of a plasmid cocktail in comparison to immune responses to vaccination with individual components [16]. In that trial, none of 31 volunteers immunized with the pentavalent pre-erythrocytic stage vaccine developed T-cell responses to more than three of the five antigens, as measured by IFN-γ ELIspot assay and none of the volunteers were protected against *Pf *sporozoite challenge [16]. A lack of protection was also noted in a study combining the *Pf*CSP recombinant protein vaccine RTS,S vaccine and recombinant *Pf*SSP2/TRAP [17]. Interference studies subsequently conducted in mice with mammalian codon-optimized versions of the same five pre-erythrocytic genes plus four erythrocytic stage genes (*Pf*AMA1, *Pf*MSP1-3D7, *Pf*MSP1-FVO, and *Pf*EBA175) showed significant inhibition of antigen-specific T-cell and antibody responses when nine plasmid DNA vaccines were administered as a single cocktail [18]. In vitro expression studies indicated that the inhibition was occurring at the level of mRNA [19]. In a series of plasmid competition experiments, the nine codon-optimized *P. falciparum *plasmid DNA vaccines were assessed for immunogenicity and multi-antigen compatibility, by being tested individually as the nine-valent cocktail, and as a series of eight-valent cocktails in which each single antigen was removed from the cocktail in turn to see if the remaining plasmids were released from an interference effect [18]. Those data led to the down-selection of a five antigen combination of three pre-erythrocytic stage vaccines encoding the *P. falciparum *(3D7 strain) of CSP [20,21], SSP2/TRAP [22,23] and LSA1 [24,25] as well as two erythrocytic stage antigens, AMA1 [26,27], and MSP1-42 [28,29] in which plasmid interference appears to be minimized. This antigen combination has been called CSLAM.

In contrast to recombinant protein approaches, molecular vaccines such as plasmid DNA and recombinant attenuated live viruses rely on the mammalian host's cellular machinery to translate the injected genetic material to produce the foreign protein(s) that is expressed in the correct conformation for recognition by the host immune system [30]. Thus, molecular vaccines offer the potential for the construction of multi-antigen immunogens and for activating all arms of the immune system (both antibody and cellular) to confer broad protection against pathogen challenge. The initial emphasis was on plasmid DNA vaccines as a core technology, because of their simplicity of design, ease of modification, combination (potential for multi-antigen vaccination), and manufacturing, and potential for generating the CD8+ T-cell responses required for protection against an intracellular pathogen such as *Plasmodium *[31,32]. In the malaria model, previous studies have established the capacity of DNA vaccines encoding *Plasmodium *antigens to induce CD8^+ ^CTL and IFN-γ responses and protection against sporozoite challenge in mice [15,33-36] and monkeys [37-39]. Phase I/2a clinical trials have established the safety, tolerability and immunogenicity of DNA vaccines encoding malaria parasite antigens in normal healthy humans [3,16,40-44]. However, in multiple disease systems including malaria, DNA vaccines on their own, administered in PBS/saline, have been poorly efficacious with regard to induction of antibody responses and protective immunity in non-human primates [38,39] or humans [16,40-44]. This has led to strategies to enhance the immunogenicity of plasmid DNA, by codon optimization, vaccine/adjuvant formulations, delivery technologies and heterologous DNA prime/virus boost immunization regimens [32,45-49]. One potential DNA immune enhancement strategy is formulation of the DNA with poloxamers – surface active, water-soluble, non-ionic triblock copolymers. CRL1005 is a nonionic triblock copolymer composed of blocks of polyoxypropylene (POP) and polyoxyethylene (POE) [50-52] with a POP core of molecular weight 12 kDa and 5% POE. When formulated with a protein in a vaccine formulation [51,53], these poloxamers have been shown to have adjuvant activity. In addition to enhancing protein or inactivated vaccine-induced antibody responses in rodent models of influenza, CRL1005-formulated plasmid DNA vaccines have been shown to significantly enhance the level of antigen-specific immune responses in SIV non-human primate models after adenovirus boosting [49,50,53]. It is believed that the adjuvant properties of CRL1005 are related to its ability to aggregate into surface-activated particle but the immunological mechanism is unclear. Experimental data suggest that CRL1005-mediated immune enhancement may occur via induction of antibody and IL-2 dominated Th-1 cellular responses, as evidenced in a murine influenza model [54], or induction of IFN-γ CD8+ T cells as evidenced in a SIV non-human primate model [53].

Studies in animal models of various infectious diseases including HIV [55], influenza [56], tuberculosis [57], as well as malaria [47], have established that heterologous prime/boost strategies comprising, for example, priming with plasmid DNA and boosting with recombinant virus, are far more effective than homologous immunization regimens. In particular, priming with plasmid DNA and boosting with recombinant pox was shown to be immunogenic and protective in the *Plasmodium yoelii *rodent model [34] as well as the *Plasmodium knowlesi *non-human primate model [39]. Accordingly, using a DNA prime/poxvirus boost immunization regimen to maximize the potential for enhancing the immunogenicity and protective efficacy of DNA-based vaccines, the ability of the pentavalent CSLAM vaccine cocktail to induce antigen-specific T-cell and antibody responses was evaluated in rhesus monkeys, in comparison with *Pf*CSP alone, both in the absence or presence of the poloxamer CRL1005 aiming to enhance the immunogenicity of plasmid DNA.

## Materials and methods

### Plasmid DNA vaccines

The plasmid DNA (pDNA) vaccines used in this study, in the VR1020 backbone [58], have been described previously [18]. The *Pf*CSP (VCL-2571), *Pf*SSP2/TRAP (VCL-2576), and *Pf*AMA1 (VCL-2577) vaccines encoded full-length proteins; the *Pf*LSA1 vaccine (VCL-2559) encoded the C-terminal 281 amino acid residues (representing 65% of the nonrepeat region of full length *Pf*LSA1); and the *Pf*MSP1 vaccine (VCL-2574) encoded the 42-kDa fragment of the *Pf*MSP1 protein. All vaccines were based on the 3D7 strain of *P. falciparum*, and the coding sequence for each antigen was modified for mammalian codon usage in order to improve expression of the encoded antigen [59]. Expression of the encoded protein was confirmed *in vitro *by transient transfection of VM92 melanoma cells (kindly provided by Vical Inc.) using Lipofectamine, as described by the manufacturer (Life Technologies, Gaithersburg, MD). Protein expression was quantitated as a chemiluminescent signal by using antigen-specific monoclonal or polyclonal antibodies and a commercially obtained chemiluminescence-linked Western blot kit (Western-Light, Tropix, Bedford, Mass.) according to the manufacturer's directions. Chemiluminescent signals were detected by exposure of the processed membrane to auto radiographic film (Hyperfilm-ECL; Amersham Life Sciences Inc., Cleveland, Ohio). Plasmid DNA for immunization was produced by Vical Inc., checked for physical integrity, expression activity, concentration, and endotoxin levels, and resuspended in phosphate-buffered saline (PBS) at a concentration of 2.5 mg of pDNA/ml (500 μg of each plasmid DNA vaccine). In the *Pf*CSP alone group, 2 mg of plasmid VR1020 without antigen insert was added to 500 μg *Pf*CSP DNA to maintain the total DNA concentration at 2.5 mg/ml.

### CRL1005 formulation

The poloxamer-based vaccine formulation consisted of the non-ionic block copolymer, CRL1005 (CytRx Corporation, Los Angeles, CA) and a cationic surfactant, benzalkonium chloride (BAK, BTC 50 NF, Stepan Company, Northfield, IL), formulated with pDNA in PBS. To make the formulation, the required concentration of pDNA (to produce a final concentration of 5 mg/ml) in PBS was stirred on ice and the required amount of CRL1005 (to produce a final concentration of 7.5 mg/ml) was added using a positive displacement pipette. The solution was stirred on ice until the poloxamer dissolved and then the required concentration of benzalkonium chloride dissolved in PBS was added (to produce a final concentration of 0.3 mM). The solution was then cycled through the cloud point (4 to 25°C) several times to ensure homogeneity, diluted with PBS to a final pDNA concentration of 2.5 mg/ml, and then filter sterilized at 4°C and stored frozen (-30°C). Prior to injection, the vaccine formulation was thawed at ambient temperature, stored at room temperature, and administered within 6 hours of preparation.

### Recombinant Poxvirus

ALVAC-*Pf7 *(virus # vCP1305, stock # N37) is a highly attenuated canary poxvirus with seven *P. falciparum *genes (native codon sequence) inserted into its genome: *Pf*CSP and *Pf*SSP2/TRAP (sporozoite stage); *Pf*LSA1 (liver stage); *Pf*AMA1, *Pf*MSP1, and *Pf*SERA (blood stage), and *Pf*s25 (sexual stage). The recombinant virus was produced by Virogenetics Corporation (Troy, NY).

### Recombinant proteins

Recombinant *Pf*CSP [60], *Pf*LSA1 (D. Lanar, WRAIR), *Pf*AMA1 [61], and *Pf*MSP1-42 (A. Saul, MVDB) were well characterized *Pf *antigens (3D7 strain) manufactured at research grade. The recombinant proteins were used at a final concentration of 10 μg/ml as antigen for *in vitro *ELIspot and intracellular cytokine staining assays, and at a final concentration of 1 μg/ml as capture antigen for antibody ELISAs. The endotoxin levels were <5 EU/ml for recombinant *Pf*AMA1, and *Pf*MSP1; 13 EU/ml for *Pf*CSP; and 74 EU/ml for *Pf*LSA1, as indicated by the LAL testing (Cambrex BioScience Walkersville, Inc, MD).

### Synthetic peptides and peptide pools

A series of 15-mer synthetic peptides, with 10-mer overlaps, derived from *Pf*CSP (total 64 peptides), *Pf*SSP2/TRAP (total 138 peptides) and *Pf*LSA1 (total 114 peptides) were synthesized by Mimotopes (Melbourne, Australia) at > 80% purity. Individual peptides were resuspended in DMSO at a concentration of 20 mg/ml and pooled sequentially according to antigen sequence, with 22 peptides or less per pool. Peptides were used at a final concentration of 4 μg/ml for *in vitro *T cell assays. The final DMSO concentration did not exceed 0.55%.

### Immunization regimen

A total of 20 rhesus monkeys (*Macaca mulatta*), aged 5 to 10 years and between 3 and 10 kg in weight were screened for anti-*Pf*CSP antibody negativity by ELISA and randomized into four groups with five monkeys per group. At weeks 0, 4, and 8, monkeys were immunized with 500 μg *Pf*CSP plasmid DNA either in PBS (monkey RZc7, RHc7, RLw6, RSf7, Rli6) or CRL-1005 (monkey RLf7, RUm6, ROr6, RAs6, RDr6), or 500 μg of each CSLAM plasmid DNA either in PBS (monkey RDc7, RCf7, RWa7, RYi6, RVi6) or formulated in CRL1005 (PH1019, ROk6, ROa7, RQk6, RBsG) in a total volume of 1 ml, administered intramuscularly in single sites in the rectus femoris muscle using a 22-gauge needle. Subsequent immunizations were administered to the same muscle on alternating sides of the animal. Twelve weeks after the last pDNA immunization, animals were boosted by intramuscular administration of 2 × 10^8 ^pfu of ALVAC-*Pf7*. All immunizations were carried out at the Emory Vaccine Center at the Yerkes National Primate Research Center, Emory University, GA. All experiments were conducted in compliance with the Animal Welfare Act and with Emory University Institutional Animal Care and Use Committee approvals in accordance with the principles set forth in the "Guide for the Care and Use of Laboratory Animals", Institute of Laboratory Animal Resources, National Research Council, and National Academy Press, 1996. Sera and peripheral blood mononuclear cells were collected pre-immunization and at 4 weeks post each immunization for assessment of immunogenicity. The animals were monitored and evaluated daily for general behaviour, level of activity, and visible side effects or adverse reactions. The injection sites were examined closely by the veterinary clinician each time the animals were sedated for designated bleedings and injections. Close observations included analysis of skin for warmth, erythema and edema/swelling, and muscle induration. The clinical veterinarian examining the animals and injection sites was blinded to which vaccine formulation had been given. Analysis of hematology and clinical chemistry of the different animals was performed on blood samples drawn at pre-bleed, immunization and boosting time points. Hematology analysis consisted of an evaluation of white blood cells, erythrocytes, hemoglobin, hematocrit, mean corpuscular volume and platelets values. Analysis of clinical chemistry consisted of glucose, blood urea nitrogen, creatinine, protein, albumin, alkaline phosphatase, serum glutamic pyruvic transaminase, serum glutamic oxaloacetic transaminase, amylase and creatine phosphokinase determinations.

### IFN-γ ELIspot assay

The assay for rhesus IFN-γ was modified from a previously described method [62]. In brief, 96-well PVDF plates (Millipore Corporation, Bedford, MA) were coated with 100 μl/well of anti-human IFN-γ mAb (clone GZ-4, BenderMedSystems, Burlingame, CA) at a concentration of 5 μg/ml in 1× PBS and incubated overnight at 4°C. Plates were washed six times with RPMI 1640 medium and blocked with 200 μl/well of 10% FCS-RPMI 1640 medium (R10) for at least 2 hr at 37°C. After blocking, the plates were washed once with R10, and 100 μl of 2 × 10^6 ^PBMC (200,000 cells/well) and 100 μl of stimulant in R10 were added per well, in quadruplicate. Plates were incubated for 18 hrs at 37°C in an atmosphere of 5% CO_2_. Plates were then washed six times with 1× PBS in the presence of 0.5% Tween 20 (PBS-T) 100 μl/well of 1 μg/ml biotinylated anti-IFN-γ (clone 7-B6-1, MabTech, Sweden) added per well, and the plates incubated for 1 hr at 37°C. Plates were washed six times with 1× PBS-T, and 100 μl/well streptavidin-alkaline phosphatase conjugate (MabTech, Sweden) was added at 1:1000 dilutions in PBS. After 1 hr incubation at room temperature, plates were washed six times with 1× PBS-T followed by three times with PBS, and developed with AP conjugate substrate kit, (BioRad Laboratories, Hercules, CA) according to the manufacturer's instructions. After 15 min, the plates were rinsed extensively with dH_2_O to stop the colorimetric reaction, dried and stored in the dark. The IFN-γ spot-forming cells (SFC) were numerated using a high-resolution automated ELIspot reader (Carl Zeiss Vision, Germany). Responses were expressed as the mean number of SFC per million cells in quadruplicate wells. Responses, to both protein and peptide pools, were classified as positive if (1) the net SFC (mean SFC in experiment antigen wells – mean SFC in medium wells) was > 25 SFC per million PBMC, and (2) the stimulation index (ratio of mean SFC in experimental peptide wells to mean SFC in medium wells) was > 2. In some cases, responses in cells collected before immunization to a specific immunogen met the criteria of positivity as defined above. In that case, responders post immunization were classified as positive if the magnitude of net ELIspot was > 2-fold that of the pre-immunization response.

### Intracellular cytokine staining

All reagents for the intracellular cytokine staining were purchased from Becton Dickinson ImmunoCytometry Systems (San Jose, CA). A total of 0.5–1.0 × 10^6 ^PBMC in 100 μl R10 medium were plated per well in U-bottomed 96-well plates, in the presence of 1 μg/ml anti-human CD28 (Clone CD28.2) and 1 μg/ml anti-human CD49d (clone 9F10) antibodies with or without 100 μl antigen (total 200 μl/well). GolgiPlug was added at 1 μl/well at 2 hrs after the start of incubation, and the plates were then incubated an additional 14 hrs at 37°C in an atmosphere of 5%CO_2_. Plates were spun at 1,200 rpm for 5 min, the supernatant flicked, the cell pellet resuspended by gentle vortexing, and the cells washed twice in FACS buffer. The cells were stained with 20 μl anti-CD4-PerCP-Cy5.5 (clone L200), and 5 μl anti-CD8-PE-Cy7 (clone RPA-T8) in a final volume of 100 μl FACS buffer for 45 min on ice in the dark. After the surface staining, cells were washed with FACS buffer twice, gently resuspended, and permeabilized in 100 μl CytoFix/CytoPerm buffer for 20 min. Cells were washed again and then stained with anti-IFN-γ-FITC (clone B27) and anti-IL2-APC (clone MQ1-17H12) for 45 min on ice in the dark. Cells were washed twice with CytoPerm wash buffer, resuspended in 200 μl of FACS buffer, and stored at 4°C prior to analysis. Samples were analysed using the FACSCalibur™ flow cytometer (Becton Dickinson Immunocytometry Systems, San Jose, CA) and CellQuest software. The expression level of intracellular cytokines was presented as the percentage of stained cells in gated cell populations of either CD4+ or CD8+ cells, corrected for background responses in the absence of antigen. The non-specific background was typically less than 0.05%.

### Antibody assays

Pre- and post-immunization serum samples were serially diluted two-fold and assayed in parallel for anti-*Plasmodium *antibodies. Parasite-specific antibodies were assayed by the indirect fluorescent-antibody test (IFAT) [63] against air-dried *P. falciparum *(strain NF54 ~ 3D7 clone) sporozoites or parasitized erythrocytes. IFAT results were reported as the endpoint dilution, representing the last serum dilution at which fluorescence was scored as positive. Antigen-specific antibodies were assayed by the enzyme-linked immunosorbent assay (ELISA) [63,64] against recombinant *Pf*CSP (0.5 μg/ml), *Pf*SSP2/TRAP (1.0 μg/ml), *Pf*LSA1 (1 μg/ml), *Pf*AMA1(1 μg/ml), or *Pf*MSP1 (1 μg/ml) proteins. ELISA results were reported as OD 0.5 units, representing the reciprocal of the serum dilution at which the mean OD reading was 0.5. ELISA responses were classified as positive if (1) titer of OD 0.5 units was > 25 and (2) the seroconversion index (ratio of titer post-immunization to titer pre-immunization) was > 4.

### Statistical analysis

Experimental outcomes were presented as direct results of ELIspot assays (IFN-γ spot forming cells/million splenocytes), FACS analysis (% responding cells), ELISA (antibody titers) or IFAT (antibody titers). Comparison of ELISA data and intracellular cytokine expression were done by 2-tailed student t test. ELIspot data are reported as mean +/- standard deviation of quadruplicate wells. Responses to all individual antigens, either recombinant proteins (*Pf*CSP, *Pf*LSA1, *Pf*AMA1, and *Pf*MSP1) or peptide pools (i.e. three *Pf*CSP pools, seven *Pf*SSP2/TRAPpools, six *Pf*LSA1 pools), were analysed with an analysis of variance for a repeated measures design with one between-subjects factor (vaccine group at four levels), and one within-subjects factor (time at six levels). All pair-wise comparisons of means were made with Fisher's LSD (Least Significant Difference) test. The sums of the peptide pools were analysed with the same methods. The recombinant proteins were analysed with the same methods except for the addition of one between-subjects factor (called r-protein with 4 and 5 levels, respectively, for analyses with and without *Pf*SSP2/TRAP). Fisher's exact test was used informally to conduct pair-wise vaccine group comparisons of positive responder rates. Trends over time were examined with graphs, but no formal statistical tests of significance were conducted to test for significant trend components (i.e. linear, quadratic or cubic components). The p-value considered significant was p < 0.05.

## Results

### Clinical evaluation

Animals were evaluated daily for general behaviour and the presence of any adverse reactions or clinical abnormalities, as described in Material and Methods. No unusual behaviour or adverse reactions were observed throughout the course of the trial. Haematology and clinical chemistry values remained within the normal range.

### Antigen-specific and parasite-specific antibody responses

The induction of parasite-specific and antigen-specific antibody responses post vaccination was assessed by IFAT against air-dried *Pf *sporozoites or parasitized erythrocytes, and by ELISA against each vaccine antigen component, respectively. IFAT responses were evaluated pre-immunization, after the 3^rd ^DNA immunization, and after virus boost. Positive IFAT responses against *Pf *sporozoites were detected post DNA immunization in 5/5 animals in the CSLAM/PBS immunized group but 0/5 in the *Pf*CSP/PBS immunized group (data not presented). The titer of IFAT increased approximately five-fold after virus boost (mean, 160 versus 30). No blood-stage parasite specific antibodies were detected by IFAT at any time point.

**Figure 1:**
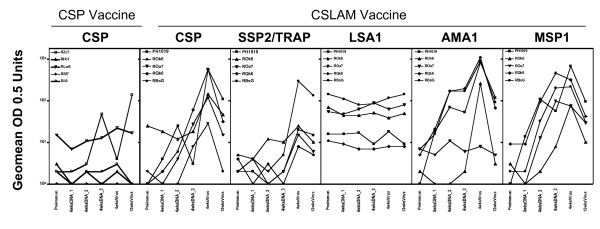
**Antigen-specific antibody responses against pre-erythrocytic antigens *Pf*CSP, *Pf*SSP2/TRAP, *Pf*LSA1 and blood stage antigens *Pf*AMA1 and *Pf*MSP1**. Sera from monkeys immunized with either CSP/PBS or CSLAM/PBS were assayed against *Pf*CSP, *Pf*SSP2/TRAP, *Pf*LSA1, *Pf*AMA1 or *Pf*MSP1 capture antigens by ELISA. Data are presented as the geometric means of titers at OD 0.5 units from individual monkeys per group. ELISA responses were classified as positive if (1) OD0.5 unit titer was > 10; and (2) the seroconversion index (ratio of titer post-immunization to titer pre-immunization) was > 4. Preimmun = preimmunization; 4wksDNA_1 = 4 wks post 1^st ^DNA immunization; 4wksDNA_2 = 4 wks post 2^nd ^DNA immunization; 4wksDNA_3 = 4 wks post 3^rd ^DNA immunization; 4wksVirus = 4 wks post ALVAC-*Pf*7 boost; 12 wksVirus = 12 wks post ALVAC-*Pf*7 boost.

To determine whether co-immunization of *Pf*CSP with four additional antigens, *Pf*SSP2/TRAP, *Pf*LSA1, *Pf*AMA1 and *Pf*MSP1 in PBS (i.e. the other antigens of the CSLAM/PBS cocktail), adversely affected the *Pf*CSP-antigen specific antibody responses, the frequency of positive responders and the magnitude of ELISA responses were compared between *Pf*CSP/PBS immunized and CSLAM/PBS immunized animals. No antigen-specific antibodies were detected after the first DNA immunization. In monkeys immunized with *Pf*CSP/PBS alone, anti-*Pf*CSP antibodies could not be detected until after third DNA immunization, when only1/5 monkeys met the criteria of positivity and remained positive after virus boost (Figure 1). In monkeys immunized with CSLAM/PBS, however, anti-*Pf*CSP antibodies were detected in 1/5 monkeys after the 2^nd ^immunization, 3/5 monkeys after 3^rd ^DNA immunization, and 5/5 monkeys after virus boost. The anti-*Pf*CSP antibody response was significantly higher in the CSLAM/PBS immunized responder monkeys as compared with *Pf*CSP/PBS immunized responders after the viral boost (p = 0.044), but not after DNA immunization (p = 0.20). In the CSLAM/PBS group, anti-*Pf*AMA1 and anti-*Pf*MSP1 antibodies were detected in 0/5 monkeys after the 1^st ^DNA immunization, 3/5 monkeys after 2^nd ^DNA immunization, 3/5(*Pf*AMA1) or 4/5 (*Pf*MSP1) after the 3^rd ^DNA immunization, and 4/5 (*Pf*AMA1) or 5/5 (*Pf*MSP1) monkeys after the viral boost. Anti-*Pf*SSP2/TRAP antibodies were detected in 2/5 monkeys post-viral boost only. No anti-*Pf*LSA1 specific antibody responses were detected at any time point. After virus boosting, antibody titers increased approximately 6-fold for *Pf*CSP, 9-fold for *Pf*AMA1, and 2-fold for *Pf*MSP1 (Figure 1). Overall, the most immunogenic antigen with regard to antibody response was *Pf*AMA1.

### P*f*CSP-specific ELIspot responses

**Figure 2:**
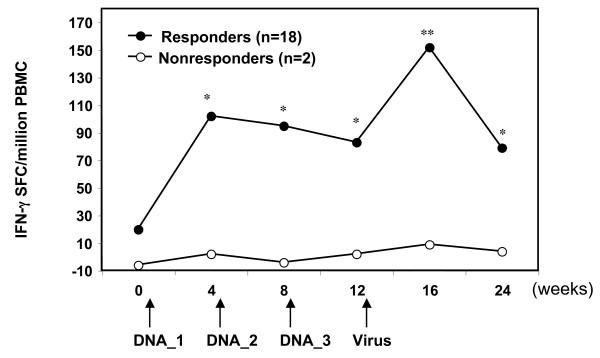
**Antigen-specific IFN-γ responses against pre-erythrocytic antigen *Pf*CSP**. PBMC from *Pf*CSP or CSLAM immunized monkeys (both PBS and CRL1005 formulations) were assayed against recombinant *P*fCSP protein by IFN-**γ **ELIspot. Results represent the mean net spot-forming cells (SFC) per million PBMC from all responders (n = 18) (solid circle) or non-responders (n = 2) (open circle) at the defined time points. DNA_1 = 1^st ^DNA immunization; DNA_2 = 2^nd ^DNA immunization; DNA_3 = 3^rd ^DNA immunization; Virus = ALVAC-*Pf*7 boost. Net SFC was calculated by correcting the SFC responses with antigen for background SFC without antigen. IFN-γ ELIspot responses were classified as positive if (1) the net SFC (mean SFC in experiment antigen wells – mean SFC in medium wells) was > 25 SFC per million PBMC, and (2) the stimulation index (ratio of mean SFC in experimental peptide wells to mean SFC in medium wells) was > 2. (*p < 0.05 and **p < 0.01).

Overall, antigen-specific IFN-γ ELIspot responses were observed in 18/20 immunized monkeys regardless of groups and immunogens, and responses were detected as early as 4 weeks after a single DNA immunization. The two non-responder monkeys were monkey RCf7 (CSLAM/CRL10005) and monkey RHc7 (CSP/PBS). The average SFC from positive responders post 1^st ^DNA immunization (mean 102 SFC/million, range 0–273 SFC/million) was approximately 5 times higher than the average SFC response before immunization (20 SFC/million, p < 0.05) (Figure 2). The ELIspot responses plateaued post 2^nd ^(mean 95, range 0–286 SFC/million) and 3^rd ^(mean 83, range 0–401 SFC/million) DNA immunizations but were boosted by ALVAC-*Pf7 *with magnitudes increasing to an average of 152 SFC/million (range 0–466 SFC/million, p = 0.005 compared to preimmunization) at 4 weeks post virus boost. The response then declined at 12 weeks post viral boost to a level similar to that prior to the boost (mean 79, range 0–268 SFC/million). A similar pattern of *Pf*CSP-specific IFN-γ ELIspot responses was observed to synthetic *Pf*CSP peptide pools, although the overall magnitude of SFC to individual peptide pools was not as great as to the recombinant protein (data not presented). These data confirm previous studies by us [37], demonstrating the immunogenicity of *Pf*CSP plasmid DNA in rhesus monkeys via detection of CTL activity at 3 weeks after the 2^nd ^DNA immunization. In that study, the *Pf*CSP DNA was native rather than mammalian codon optimized, and CTL responses could not be detected after a single DNA immunization. In other studies in the *P. knowlesi*/rhesus model, with native *P. knowlesi *antigens, no response could be detected even after three DNA immunizations [38,39].

### Comparison of P*f*CSP-specific IFN-γ ELIspot responses between P*f*CSP and CSLAM groups

To determine whether co-immunization of *Pf*CSP with *Pf*SSP2/TRAP, *Pf*LSA1, *Pf*AMA1, and *Pf*MSP1 adversely affected the *Pf*CSP-antigen specific T cell responses, the frequency of positive responders and the magnitude of ELIspot responses were compared between *Pf*CSP/PBS immunized and CSLAM/PBS immunized animals. There was no significant difference between the frequency of *Pf*CSP-specific IFN-γ positive responders in monkeys immunized with *Pf*CSP/PBS alone or with *Pf*CSP in the pentavalent cocktail CSLAM/PBS at anytime point, as determined by responses against both recombinant protein and synthetic peptide immunogens (Table 1). Interestingly, and consistent with the antibody data presented above, there were more *Pf*CSP responders in the group immunized with CSLAM/PBS as compared to the group immunized with *Pf*CSP/PBS alone.

**Table 1:** Frequency of positive responders to *Pf*CSP in CSP and CSLAM groups

	**Recombinant Protein**		**Synthetic Peptide Pools**	
	**CSP**	**CSLAM**	**CSP**	**CSLAM**
**Preimmunization**	0/5	0/5	0/5	0/5
**4wksDNA_1**	2/5	2/5	0/5	0/5
**4wksDNA_2**	2/5	2/5	0/5	0/5
**4 wksDNA_3**	2/5	2/5	0/5	0/5
**4wksVirus**	2/5	3/5	1/5	2/5
**12wksVirus**	2/5	4/5	2/5	4/5

**Figure 3:**
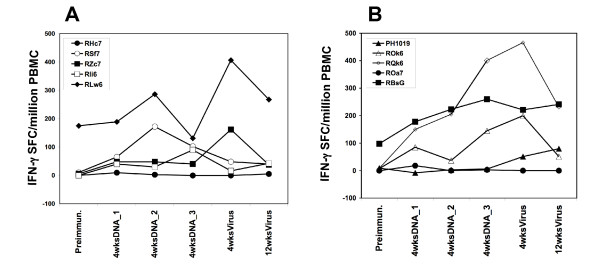
**Magnitude of *Pf*CSP-specific IFN-γ responses from *Pf*CSP/PBS and CSLAM/PBS immunized animals**. PBMC were collected at time points as defined in the legend to Figure 2 and assayed against recombinant *Pf*CSP protein by IFN-γ ELIspot. Results show the magnitude of SFC from individual monkeys immunized with either (A) *Pf*CSP/PBS or (B) CSLAM/PBS.

Similarly, there was no significant difference in the magnitude of *Pf*CSP-specific IFN-γ ELIspot responses between the *Pf*CSP/PBS and CSLAM/PBS groups (Figure 3). At four weeks post 3^rd ^DNA immunization, the SFC/million in responders to recombinant *Pf*CSP protein ranged between 145 and 401 SFC/million (mean 269 SFC/million PBMC) for the CSLAM/PBS group as compared with a range of 40–102 SFC/million (average 71 SFC/million) for the *Pf*CSP/PBS group. At 4 wks post viral boost, the magnitude of IFN-γ ELIspot forming cells was 246 SFC/million (range 51–466 SFC/million PBMC) for the CSLAM/PBS group versus 284 SFC/million (range 162–406 SFC/million PBMC) for the *Pf*CSP/PBS group.

These ELIspot data, together with the antibody data above, demonstrate that co-immunization of *Pf*CSP with two other pre-erythrocytic stage antigens (*Pf*SSP2/TRAP, *Pf*LSA1) and two erythrocytic stage antigens (*Pf*AMA1 and *Pf*MSP1) does not adversely affect the immunogenicity of *Pf*CSP. These data support the down-selection of the CSLAM antigen combination, as determined by studies in the *P. yoelii *model [18].

### CSLAM-specific ELIspot responses

**Figure 4:**
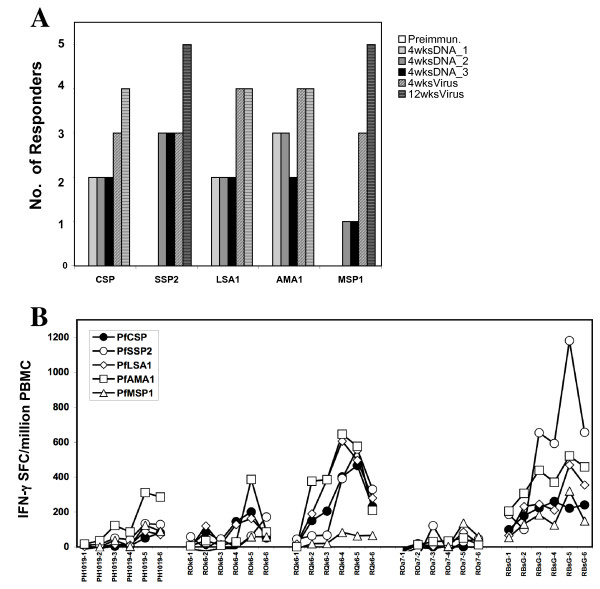
**A. Frequency of IFN-γ ELIspot responders to all CSLAM antigen components in CSLAM/PBS immunized monkeys**. Monkeys were immunized with CSLAM/PBS, and PBMC collected at time points as defined in the legend to Figure 2 were assayed against recombinant *Pf*CSP, *Pf*LSA1, *Pf*AMA1 or *Pf*MSP1 protein and *PfSSP2/TRAP *peptide pools by IFN-γ ELIspot. **B**. **Magnitude of IFN-γ ELIspot responses to all CSLAM antigen components in CSLAM/PBS immunized monkeys**. Monkeys were immunized with CSLAM/PBS and PBMC collected at defined time points were assayed against recombinant *P*fCSP, *Pf*LSA1, *Pf*AMA1 or *Pf*MSP1 protein and *Pf*SSP2/TRAP peptide pools by IFN-γ ELIspot. Time point number 1, 2, 3, 4, 5, and 6 represent time points pre-immunization, 4 wks post 1^st ^DNA immunization, 4 wks post 2^nd ^DNA immunization, 4 wks post 3^rd ^DNA immunization, 4 wks post ALVAC-*Pf*7 boost, and 12 wks post ALVAC-*Pf*7 boost, for monkeys PH1019, ROk6, RQk6, ROa7 and RBsG, respectively.

Antigen-specific responses to the other components of the CSLAM vaccine were also assessed. As shown in Figure 4A, antigen-specific IFN-γ ELIspot response to all other components of CSLAM/PBS vaccine (*Pf*LSA1 recombinant protein and synthetic peptides, *Pf*SSP2/TRAP synthetic peptide, *Pf*AMA1 recombinant protein, and *Pf*MSP1 recombinant protein) were induced by DNA immunization and were boosted by ALVAC-*Pf7 *(peptide data other than *Pf*SSP2/TRAP not presented). IFN-γ responses to *Pf*SSP2/TRAP and *Pf*MSP1 were detected in 5/5 CSLAM/PBS immunized monkeys, and responses to *Pf*CSP, *Pf*LSA1 and *Pf*AMA1 were detected in 4/5 monkeys. Positive responses to *Pf*LSA1 and *Pf*AMA1 were observed after the 1^st ^DNA immunization, as noted for *Pf*CSP, whereas positive responders to *Pf*SSP2/TRAP and *Pf*MSP1 were detected only after the 2^nd ^DNA immunization. After the ALVAC-*Pf7 *boost, at least 3/5 monkeys met the defined criteria of positive responses for each immunogen (Figure 4A).

The magnitude of vaccine-induced IFN-γ ELIspot responses in the 5 CSLAM/PBS immunized monkeys to each of the CSLAM components is shown in Figure 4B. The most robust response, at 4 weeks post virus boost, was against *Pf*AMA1 (in 4/5 monkeys); this response was not significantly different from the anti-*Pf*LSA1 response (p > 0.05) but was significantly higher than the *Pf*CSP- and *Pf*MSP1-specific responses (p < 0.05). Of the five antigens tested, *Pf*MSP1 was the least immunogenic for T cell responses at all time points. For all antigens and all responder monkeys (with the exception of one *Pf*CSP immunized monkey), IFN-γ ELIspot responses were boosted byALVAC-*Pf*7 virus. All boosted responses decreased within 12 weeks post viral boost to a level similar to that prior to the boost.

Consistent with the known genetic restriction of T cell responses to *Plasmodium *proteins [65], heterogeneity of IFN-γ responses amongst individual monkeys was noted. The same monkey did not respond to all antigens, and different monkeys responded to different antigens (Figures 4A and 4B). For example, at four weeks post virus boost, two monkeys responded to five antigens, one to four antigens, one to two antigens, and one to only one antigen. The most robust IFN-γ responses to all five antigens were detected in monkey RBsG in the CSLAM/PBS group; strong responses to recombinant *Pf*CSP, *Pf*AMA1, *Pf*SSP2/TRAP, and *Pf*LSA1 but not *Pf*MSP1, were detected in monkey RQk6; monkeys PH1019 and ROk6 exhibited strong responses to *Pf*AMA1 but weak response to the other four antigens; and monkey ROa7 had a poor response to *Pf*MSP1 and no response to the other four antigens.

**Figure 5:**
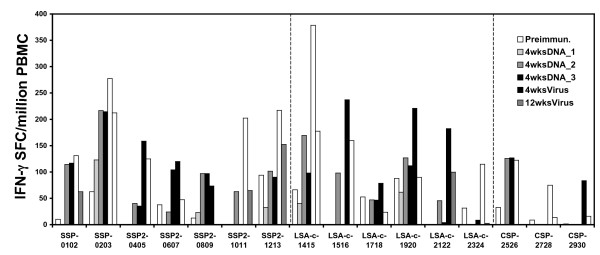
**IFN-γ responses to individual peptide pools derived from *Pf*CSP, *Pf*SSP2/TRAP or *Pf*LSA1**. PBMC from monkey RBsG (CSLAM/PBS) collected at time points as defined in the legend to Figure 2 were assayed against pools of synthetic peptides spanning the complete sequences of *Pf*CSP, *Pf*SSP2/TRAP or *Pf*LSA1 by IFN-γ ELIspot.

For those antigens for which synthetic peptides spanning the complete antigen were available (*Pf*CSP, *Pf*SSP2/TRAP, and *Pf*LSA1), responses to multiple peptide epitopes were detected. Representative data for responses in monkey RBsG, the most reactive monkey in the CSLAM/PBS group, are presented in Figure 5. Among the three antigens (and consistent with the recombinant protein reactivity reported above), the most robust IFN-γ responses were detected to *Pf*LSA1, with positive responses to five of the six peptide pools. The most N-terminal pool LSA1–1415 (residues 1–84) was the most immunogenic, with an average of 380 SFC/million PBMC at 4 wks post ALVAC-*Pf7 *boost. The magnitude of IFN-γ responses in three of the other four *Pf*LSA1 peptide pools was comparable, and the least reactive pool was LSA1–1718 (residues 132–236). Similarly, for *Pf*CSP, the most immunogenic peptide pool was the most N-terminal pool CSP-2526 (residues 1–184), although the magnitude of responses to *Pf*CSP peptide pools was lower than that for the other two antigens. The frequency and magnitude of responses to the *Pf*SSP2/TRAP peptide pools were variable; the most immunoreactive pool was residues 44–128, with an average of 277 SFC/million PBMC at 4 wks post viral boost.

These data establish that T cell responses to each component of the CSLAM/PBS vaccine were detected in rhesus monkeys following DNA immunization and were boosted by ALVAC-*Pf7*.

### Effects of CRL1005 formulation on immunogenicity of P*f*CSP and CSLAM vaccines

**Figure 6:**
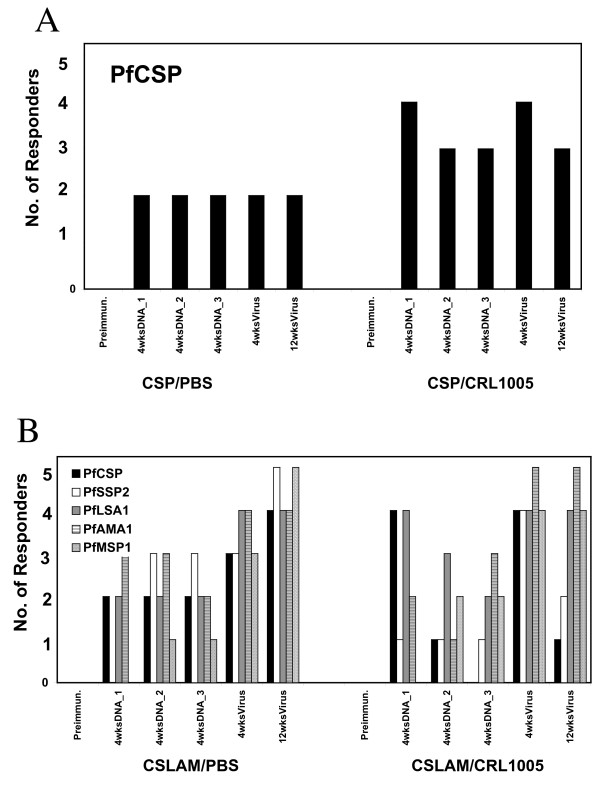
**Effect of CRL1005 formulation on frequencies of IFN-γ ELIspot responders in (A) *Pf*CSP or (B) CSLAM immunized monkeys**. Monkeys were immunized with either *Pf*CSP or CSLAM in PBS (open circle) or formulated in CRL1005 (solid circle). PBMC were collected at time points as defined in the legend to Figure 2 and assayed against recombinant *Pf*CSP, *Pf*LSA1, *Pf*AMA1 or *Pf*MSP1 protein and *Pf*SSP2/TRAP peptide pools by IFN-γ ELIspot. Data represent the number of animals with responses that met the defined criteria of positivity (SFC = 25 and SI = 2).

**Figure 7:**
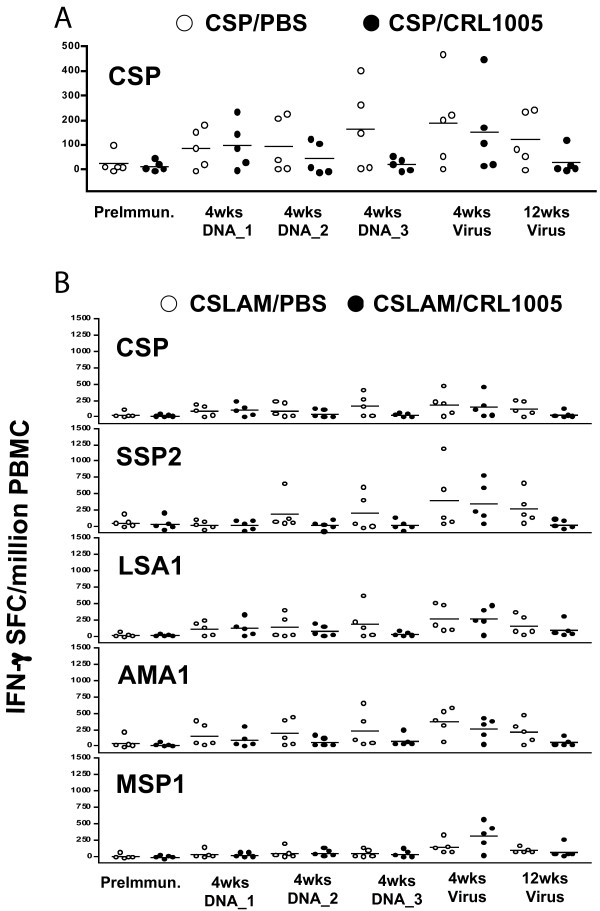
**Effects of CRL1005 formulation on the magnitude of IFN-γ ELIspot responses in (A) *Pf*CSP/PBS versus *Pf*CSP/CRL1005 or (B) CSLAM/PBS versus CSLAM/CRL1005 immunized monkeys**. Monkeys were immunized with either *Pf*CSP or CSLAM in PBS (open circle) or formulated in CRL1005 (solid circle). PBMC were collected at time points as defined in the legend to Figure 2 and assayed against recombinant *P*fCSP, *Pf*LSA1, *Pf*AMA1 or *Pf*MSP1 protein and *Pf*SSP2/TRAP peptide pools by IFN-γ ELIspot. Data represent the magnitude of IFN-γ responses specific for *P*fCSP, *Pf*SSP2/TRAP, *Pf*LSA1, *Pf*AMA1 or *Pf*MSP1, for each monkey.

To investigate the capacity of CEL1000 to enhance the immunogenicity of plasmid DNA, the frequency and magnitude of antigen-specific IFN-γ T cell responses, and parasite-specific and antigen-specific antibody responses, in monkeys administered *Pf*CSP or CSLAM formulated in CRL1005, were evaluated in comparison with those in PBS. The frequencies of responders to *Pf*CSP in the *Pf*CSP/PBS and CSP/CRL1005 immunized monkeys, and the frequencies of responders to each CSLAM antigen in the CSLAM/PBS and CSLAM/CRL1005 group, are presented in Figure 6. The respective magnitudes of responses are presented in Figure 7. Overall, for *Pf*CSP as well as the other CSLAM antigens, there was no measurable effect of CRL1005 formulation on enhancement of either frequency (Figures 6A and 6B) or magnitude (Figure 7) of IFN-γ responses.

Likewise, there was no apparent effect of CRL1005 formulation on the frequency or magnitude of antibody responses to *Pf*CSP in *Pf*CSP immunized animals, nor to any of the CSLAM antigen components in CSLAM immunized monkeys (Additional File 1).

### Intracellular IFN-γ and IL-2 expression by CD4+ and CD8+ T cells

**Figure 8:**
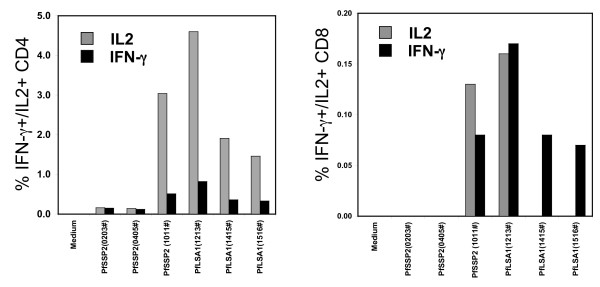
**Intracellular IFN-γ and IL-2 expression by CD4+ or CD8+ T cells**. PBMC from monkey R immunized with CSLAM/PBS, collected at preimmunization and 4 weeks post virus boost time points, were assayed against *Pf*SSP2/TRAP, or *Pf*LSA1 peptide pools by intracellular cytokine staining. Data represent the percentage of CD4+ or CD8+ IFN-γ and IL-2 producing T cells. Note differences in the y-axis scales between CD4+ and CD8+ responses.

The ELIspot assay as reported here measures the total number of cytokine secreting cells within a bulk cell population and does not allow discrimination between the responding cell phenotype(s). Therefore, to determine the phenotypes of IFN-γ-producing cells, the intracellular expression of IFN-γ and IL-2 for CD4+ and CD8+ T cell subsets, in CSLAM immunized monkeys was measured. Based on cell availability, PBMC from three monkeys (ROa7, RQk6, RBsG), collected at preimmunization and four weeks post virus boost time points, were analysed against peptide pools derived from *Pf*CSP, *Pf*SSP2/TRAP and *Pf*LSA1. Figure 8 shows the result of this analysis from the monkey RBsG, for *Pf*SSP2/TRAP and *Pf*LSA1. Peptide pools *Pf*SSP2/TRAP-1011 (residues 396–495), *Pf*SSP2/TRAP-1213 (residues 484–562), *Pf*LSA1–1415 (residues 1–84) and *Pf*LSA-1516 (residues 44–143) preferentially induced CD4+ IL-2 responses, with low level CD4+IFN-γ responses. All four pools also induced low level CD8+ IFN-γ responses, and two of them induced low level CD8+ IL-2 responses (Figure 8). These data indicate that the CSLAM DNA vaccines preferentially induce CD4+ T cell cytokine responses, rather than CD8+ T cell responses, and that IL-2 responses predominate over IFN-γ responses. The data reported above describing the correlation of summed IFN-γ responses to synthetic peptide overlaps and IFN-γ responses to the recombinant protein also suggested that the IFN-γ responses were mediated by CD4+ T cells, rather than CD8+ T cells. Taken together, these data indicate that CD4 T cells, rather than CD8+ T cells, are primarily responsible for the antigen-specific IFN-γ responses detected by ELIspot, at least under the conditions evaluated herein.

## Discussion

A multi-stage multi-immune response vaccine is designed to prevent *Plasmodium *infection by killing the majority of developing parasites in the liver, and also to prevent severe disease and death should break-through blood stage infections occur. Recent efforts are aimed at the induction of robust immune responses directed against five well characterized *P. falciparum *antigens: three pre-erythrocytic stage proteins that are expressed by irradiated sporozoites in infected hepatocytes (*Pf*CSP, *Pf*SSP2/TRAP, *Pf*LSA1), and two proteins expressed in the extracellular phase of the erythrocytic stage of the life cycle (*Pf*AMA1 and *Pf*MSP1). There is evidence that all of these antigens contribute to the immunity of irradiated sporozoite immunized volunteers or naturally acquired immunity, and immunization with each, or with its murine/primate malaria ortholog, has induced some degree of protection in various animal models and, in the case of *Pf*CSP, in humans. Herein, the general safety and immunogenicity of this pentavalent CSLAM vaccine cocktail has been evaluated in rhesus monkeys. Animals were primed with *Pf*CSP plasmid DNA or CSLAM plasmid DNA vaccines in PBS or formulated with CRL1005, and boosted with ALVAC-*Pf7*. Cell-mediated immune responses were evaluated by IFN-γ ELIspot and intracellular cytokine staining, using recombinant proteins and pools of overlapping synthetic peptides. Antigen-specific and parasite-specific antibody responses were evaluated by ELISA and IFAT, respectively.

Antigen-specific IFN-γ ELIspot and ELISA responses were detected to all CSLAM antigen components following immunization with either DNA/PBS or DNA/CRL1005. No antigen interference was observed for the frequency or magnitude of *Pf*CSP-specific T cell or antibody responses in animals receiving CSLAM as compared to *Pf*CSP alone. On the contrary, a trend of enhanced T cell and antibody responses to *Pf*CSP was noted in the CSLAM immunized animals. These data support the down-selection of the CSLAM antigen combination, contrasting with previous results of multi-antigen plasmid DNA mixtures *in vitro *and *in vivo *in mice [18] and humans [16]. These data suggest that further preclinical and clinical evaluation of CSLAM based vaccines is warranted.

It is noteworthy that this CSLAM antigen combination comprises antigens from both pre-erythrocytic (n = 3) and erythrocytic (n = 2) stages of the *Plasmodium *parasite life cycle. Thus, studies also demonstrate the potential for a multi-stage multi-immune response vaccine. The well established dichotomy of the immune system, whereby induction of robust T cell responses may be compromised by the simultaneous induction of robust antibody responses, and *vice versa *[66] previously has been a concern.

Amongst the five antigen components of CSLAM, *Pf*AMA1 appeared to be the most immunogenic, as evidenced by the frequency and magnitude of antigen specific T cell and antibody responses. *Pf*AMA1 is an antigen expressed by the blood-stage merozoite (in the micronemes and at the surface of the merozoite) [20] and pre-erythrocytic (liver) [67] stages. It is a primary candidate for a blood stage malaria vaccine designed to induce *Pf*AMA1 specific antibody responses [68,69]. However, to date, AMA1-specific T cell responses have been poorly characterized. These data suggest that *Pf*AMA1 should be considered as a prime candidate antigen also for vaccines designed to induce T cell responses, or both antibody and T cell responses.

Antigen-specific cellular immune responses, as measured by the IFN-γ ELIspot, were detected as early as 4 weeks after the first DNA immunization in nearly half of the monkeys, regardless of the immunogen. The data also indicated that DNA induced T cell responses appear earlier than antibodies, since T cell responses to most antigens assayed could be detected after a single DNA immunization, whereas antibody responses were detected only after three doses of DNA and then only to some of the antigens. This report represents the first demonstration that *P. falciparum *antigen-specific T cell response can be induced in rhesus monkeys after single dose of plasmid DNA, and one of the few reports of the immunogenicity of a single dose of plasmid DNA in any system [70]. In previous studies in rhesus monkeys, the earliest time point at which *P. falciparum *specific T cell responses could be detected was three weeks after two DNA immunizations (500 μg/antigen/dose), where CTL responses against *Pf*CSP, *Pf*LSA1, *Pf*Exp1, and *Pf*LSA1 were detected [37]. In humans, *Pf*CSP-specific CD8+ CTL responses could be detected in 0/5 and 2/5 volunteers immunized with 500 μg *Pf*CSP DNA at 2 wks after the 2^nd ^or 3rd immunization, respectively; and in 4/5 and 2/4 volunteers immunized with 2500 μg *Pf*CSP DNA at 2 wks after either the 2^nd ^or 3^rd ^immunization [40]. IFN-γ ELIspot responses could be detected in 14/31 volunteers immunized with 3 doses of 500 μg *Pf*CSP DNA (as part of a 5-plasmid mixture of *Pf*CSP, *Pf*SSP2/TRAP, *Pf*LSA1, *Pf*LSA3 and *Pf*Exp1) [16] and in 9/14 volunteers after either the 2^nd ^or 3^rd ^immunization with 2500 μg *Pf*CSP DNA [41].

In previous studies in the *P. knowlesi*/rhesus model, no T cell responses could be detected to any of four *P. knowlesi *antigens (*Pk*CSP, *Pk*SSP2, *Pk*AMA1, *Pk*MSP1) by IFN-γ ELIspot even after three doses of plasmid DNA [38,39]. This may relate to differences in antigen processing and presentation and/or host factors. In the SIV/rhesus non-human primate model, T cell responses were reported in some of the monkeys after the 2^nd ^DNA immunization but in most after the 3^rd ^dose [71], whereas antibody responses could be detected after one dose of DNA [72]. In cynomolgus monkeys, Ebola antigen-specific antibody responses were detected after the 3^rd ^DNA immunization but T cell proliferation was not detected until after a virus boost [73,74].

First generation DNA vaccines on their own, administered in PBS, have proved to be suboptimal in several non-human primate models and human clinical trials, highlighting the need for more effective immune enhancement strategies for plasmid DNA vaccines [45-48,75]. Data in the *P. falciparum*/rhesus non-human primate model reported here show that CRL1005 formulation had no apparent effect on vaccine-induced T cell responses or antibody responses, either before or after viral boost. A similar outcome was noted in mice (M. Sedegah, in preparation). These data contrast with results in other systems, where CRL1005 formulation of inactivated viral vaccines [50], recombinant protein vaccines [51,53] or plasmid DNA vaccines [49,54] resulted in a substantial enhancement of antigen-specific immune responses. In a CMV mouse immunogenicity model, CRL1005 provided restoration of both antibody and IFN-γ ELIspot responses in a multivalent vaccine [49]. It is unclear why CRL1005 does not have a detectable effect or restoration in the malaria model, but it is possible that the effect could be antigen dependent.

The studies also provided preliminary information on the effector cells and cytokines preferentially induced in the *Plasmodium *rhesus model by DNA prime/virus boost immunization. Data indicated that CD4+IL-2+ responses were more predominant than CD8+ T cell responses. This contrasts with results of comprehensive studies in murine models identifying CD8+ IFN-γ as the predominant immune effector mechanism in irradiated sporozoite and plasmid DNA vaccine induced protection [76-80]. Since these data have important implications with regard to the relevance of the rhesus *Macaca mulatta *model for evaluation of *Plasmodium *vaccines [81,82], additional studies are necessary to elucidate the *Plasmodium *vaccine-induced immune effector mechanisms in rhesus. It is important to note, however, that the results show that *Plasmodium *antigen specific CD8+ IFN-γ responses were detected in rhesus only in the presence of detectable CD4+ T cell responses, consistent with the profile of CD4+ T cell dependent CD8+ Type 1 responses observed in malaria DNA vaccine studies in humans as measured by T cell depletion and enrichment ELIspot assays and RT-PCR [44,41,40]. It has been proposed [41] that CD4+ T cells function in a bystander helper capacity for CD8+ T cell production of IFN-γ.

In summary, the immunogenicity of the CSLAM pentavalent pre-erythrocytic and erythrocytic stage antigen combination has been established in rhesus monkeys by the induction of both T cell and antibody responses to each antigen component of the multi-antigen mixture in the apparent absence of antigen interference. The two most immunogenic antigens of those tested were *Pf*AMA1 and *Pf*SSP2/TRAP. Using conventional measures of antibody and T-cell responses, CRL1005 formulation does not enhance the immunogenicity of plasmid DNA vaccines, in the rhesus non-human primate model of malaria. The presented studies also demonstrate general safety and the potential for multi-valent multi-stage and multi-immune response *Pf *vaccines based on rational antigen selection and combination. These results suggest that clinical evaluation of the CSLAM antigen combination is warranted, but that further formulation development to increase the immunogenicity of DNA encoded antigens may be required before proceeding to the clinic with DNA-based vaccines against malaria.

## Abbreviations

*CSLAM: Pf*CSP, *Pf*SSP2/TRAP, *Pf*LSA1, *Pf*AMA1 and *Pf*MSP1–42; ELISA, enzyme-linked immunosorbent assay; IFAT, indirect fluorescent-antibody test; *Pf, Plasmodium falciparum*.

## Authors' contributions

DLD, DCK and DJC, conceived and designed the study, and contributed to the supervision and execution of the research. DLD and GJ wrote the manuscript. GJ was responsible for, and participated in, the execution of the T cell immunology assays. YC was responsible for the execution of the antibody assays. MFB, GB, NR, HG, SA and VF performed the immunology assays. NBP facilitated the transfer and acquisition of reagents and samples and assisted in project coordination and management. MRG, AM, ES, and IC were responsible for the execution of the nonhuman primate studies. AG and DCK were responsible for the provision and formulation of CRL1005. DEL, AS, and LBM provided reagents for assay. CRM assisted with statistical analysis. KG assisted with construction of the vaccines. WRW assisted with vaccine preparation and sample processing. All authors read and approved the manuscript before submission.

## Supplementary Material

Additional file 1 Effects of CRL1005 formulation on the magnitude of antibody responses in (A) *Pf*CSP/PBS versus *Pf*CSP/CRL1005 or (B) CSLAM/PBS versus CSLAM/CRL1005 immunized monkeys. Monkeys were immunized with either *Pf*CSP or CSLAM in PBS (open circles) or formulated in CRL1005 (solid circles). Sera from immunized monkeys were assayed against *Pf*CSP, *Pf*SSP2/TRAP, *Pf*LSA1, *Pf*AMA1 or *Pf*MSP1 capture antigens by ELISA. Data are presented as the geometric means of titers at OD 0.5 units from individual monkeys per group. ELISA responses were classified as positive if (1) OD0.5 unit titer was > 10; and (2) the seroconversion index (ratio of titer post-immunization to titer pre-immunization) was > 4. Preimmun = preimmunization; 4wksDNA_1 = 4 wks post 1^st ^DNA immunization; 4wksDNA_2 = 4 wks post 2^nd ^DNA immunization; 4wksDNA_3 = 4 wks post 3^rd ^DNA immunization; 4wksVirus = 4 wks post ALVAC-*Pf*7 boost; 12 wksVirus = 12 wks post ALVAC-*Pf*7 boost. Click here for file
