# Supplementary figures and images for: Induction of multi-antigen multi-stage immune responses against Plasmodium falciparum in rhesus monkeys, in the absence of antigen interference, with heterologous DNA prime/poxvirus boost immunization
Source: Malar J. 2007 Oct 9;6:135. doi: 10.1186/1475-2875-6-135 (PMC2147027; doi:10.1186/1475-2875-6-135)

Geomean OD 0.5 Units

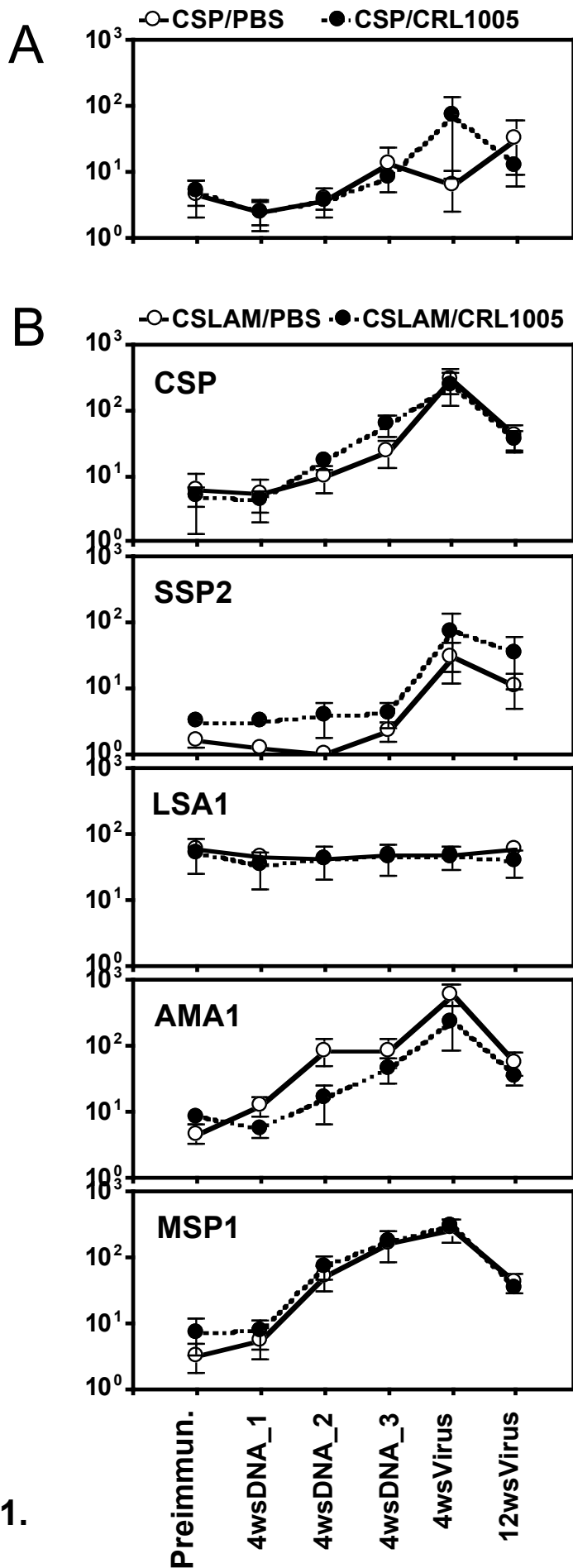

Supplementary Figure 1.

Supplement: Additional file 1 — Effects of CRL1005 formulation on the magnitude of antibody responses in (A) PfCSP/PBS versus PfCSP/CRL1005 or (B) CSLAM/PBS versus CSLAM/CRL1005 immunized monkeys. Monkeys were immunized with either PfCSP or CSLAM in PBS (open circles) or formulated in CRL1005 (solid circles). Sera from immunized monkeys were assayed against PfCSP, PfSSP2/TRAP, PfLSA1, PfAMA1 or PfMSP1 capture antigens by ELISA. Data are presented as the geometric means of titers at OD 0.5 units from individual monkeys per group. ELISA responses were classified as positive if (1) OD0.5 unit titer was > 10; and (2) the seroconversion index (ratio of titer post-immunization to titer pre-immunization) was > 4. Preimmun = preimmunization; 4wksDNA_1 = 4 wks post 1st DNA immunization; 4wksDNA_2 = 4 wks post 2nd DNA immunization; 4wksDNA_3 = 4 wks post 3rd DNA immunization; 4wksVirus = 4 wks post ALVAC-Pf7 boost; 12 wksVirus = 12 wks post ALVAC-Pf7 boost. [file 1475-2875-6-135-S1.pdf]
